# Evaluating the Impact of Nurse-Led Transition Care Guidance on Care Transition Outcomes for Children With Congenital Heart Disease: A Protocol Study

**DOI:** 10.7759/cureus.81389

**Published:** 2025-03-28

**Authors:** Shramana Ray, Asha Shetty, Bhagirathi Dwibedi

**Affiliations:** 1 Nursing, All India Institute of Medical Sciences, Bhubaneswar, Bhubaneswar, IND; 2 College of Nursing, All India Institute of Medical Sciences, Bhubaneswar, Bhubaneswar, IND; 3 Pediatrics, All India Institute of Medical Sciences, Bhubaneswar, Bhubaneswar, IND

**Keywords:** caregivers, child, congenital, health transition, heart disease, nurses

## Abstract

Background

Caregivers of children with congenital heart disease (CHD) often experience stress, uncertainty, and challenges in managing their child’s care after hospital discharge. A structured transition care approach can help ease this process by improving caregivers' preparedness, awareness, and satisfaction with post-discharge care.

Objective

This study aims to develop and evaluate a nurse-led transition care guidance (TCG) program to enhance caregivers' readiness, awareness, and satisfaction during the transition from hospital to home care for children with CHD.

Method

A mixed-methods approach was used, consisting of a quasi-experimental quantitative phase to assess the effectiveness of the TCG intervention by comparing caregivers' readiness and satisfaction scores before and after implementation and qualitative focus group discussions (FGDs) to explore caregivers' unmet needs and challenges during the transition process.

Data sources include caregiver surveys, post-discharge follow-up interviews, and hospital records. Thematic analysis will be conducted on FGDs to identify key issues affecting caregivers.

Results

The intervention is expected to improve caregivers' ability to provide care, increase their understanding of medical needs, and enhance their overall satisfaction with the transition process. Qualitative findings will highlight common challenges and gaps in post-discharge support.

Conclusion

This study will contribute to the development of evidence-based transition care plans that optimize the hospital-to-home transition for children with CHD. It highlights the importance of structured, nurse-led interventions to improve post-discharge outcomes for both caregivers and patients.

## Introduction

Congenital heart disease (CHD) remains a significant contributor to childhood morbidity, necessitating meticulous care and prolonged management. The transition from hospital to home poses multiple challenges for caregivers, who must navigate intricate medication schedules, vigilantly monitor health statuses, and promptly identify early signs of complications. A prevalent issue among caregivers is the sense of inadequacy and heightened anxiety, often stemming from insufficient education on effective management strategies [1,2].​

Existing literature reveals a scarcity of structured, evidence-based interventions specifically designed to support caregivers of children with CHD during the post-discharge transition. Notably, nurse-led interventions have demonstrated efficacy in enhancing outcomes across various patient populations; however, their application within pediatric CHD care remains underexplored. For instance, a study conducted in Southern India evaluated a structured nurse-led parent discharge teaching program and found that both nurses and parents exhibited significant improvements in home care knowledge post-implementation. Additionally, there was a notable reduction in surgical site infections among children, underscoring the program's effectiveness in promoting better home care practices after cardiac surgery [3,4].

Similarly, a quasi-experimental study in Thailand assessed the impact of a discharge planning program on caregivers' behaviors for children with CHD post-heart surgery. The findings indicated that caregivers who participated in the program demonstrated significantly improved caring behaviors compared to those who received routine care, highlighting the program's potential to enhance caregiver preparedness and reduce post-discharge complications [5-7].

To address these challenges and bridge the existing gap, this study aims to develop and evaluate a comprehensive nurse-led transition care guidance (TCG) program. The primary objectives are to enhance caregiver knowledge, bolster readiness for discharge, and elevate satisfaction with the transition process. By implementing a structured TCG program, we aspire to empower caregivers with the necessary tools and confidence to manage their children's care effectively post-discharge, thereby improving overall health outcomes for children with CHD.

This study is among the first to develop and evaluate a nurse-led TCG program specifically tailored for caregivers of children with CHD. Unlike previous interventions, which focus on one-time discharge education, this study aims to establish a structured, ongoing support system that extends into the post-discharge period. By incorporating follow-up strategies, personalized guidance, and caregiver engagement, this program seeks to bridge the gap between hospital discharge and sustained home care readiness. Furthermore, this study will contribute to a framework for nurse-led pediatric transition care, which is currently underdeveloped in CHD management.

To address these challenges and bridge the existing gap, this study aims to develop and evaluate a comprehensive TCG program. The primary objectives are to enhance caregiver knowledge, bolster readiness for discharge, and elevate satisfaction with the transition process. By implementing a structured TCG program, we aspire to empower caregivers with the necessary tools and confidence to manage their children's care effectively post-discharge, paving the way for an evidence-based, scalable model for pediatric CHD transition care.

Objectives

The primary objectives of this study are to identify the challenges and unmet needs faced by caregivers during the transition of children with CHD from hospital to home; develop a nurse-led TCG program tailored to address these identified needs; evaluate the effectiveness of the TCG intervention in improving caregiver knowledge, readiness for discharge, and satisfaction.

The secondary objectives are to assess caregiver confidence in managing care tasks, the number of post-discharge complications, and the impact of the intervention on hospital readmission rates.

## Materials and methods

This study will have a mixed-methods design, incorporating both qualitative and quantitative approaches to address the research questions. The qualitative phase will involve focus group discussions (FGDs) with caregivers, nurses, and physicians to understand the challenges and unmet needs faced by caregivers during the transition of children with CHD from hospital to home. Multi-stakeholder opinion on transition care will help identify the gaps in caregiver preparedness and inform the development of the nurse-led TCG program. The qualitative data will be analyzed using thematic analysis, which will allow the identification of common themes and patterns in the experiences and challenges reported by the participants.

In the quantitative phase, a quasi-experimental design with a pre-test and post-test control group will be used to evaluate the effectiveness of the nurse-led TCG program. The intervention group will receive the TCG program, which will include pre-discharge education sessions, personalized discharge care guides, and telephonic follow-up support at specified intervals post-discharge. The control group will receive standard discharge instructions without the additional components of the TCG program.

The study will take place in the pediatric care units of a tertiary care hospital. The participants will include caregivers of children diagnosed with congenital heart disease who are preparing for discharge. Inclusion criteria will include caregivers who are the primary decision-makers and care providers for the child and caregivers of children with congenital heart disease who are scheduled for discharge from the pediatric care unit. Exclusion criteria will include caregivers who have limited language proficiency, caregivers who are unable to attend the intervention sessions, and caregivers who are unable to commit to the follow-up process due to logistical or other reasons.

The control group will receive the standard discharge care provided by the hospital, which includes routine discharge instructions given by healthcare providers before the child is discharged. This typically consists of verbal guidance on medication administration, follow-up appointments, basic home-care instructions, and emergency signs to watch for. However, caregivers in the control group will not receive the additional structured components of the nurse-led TCG program. Specifically, they will not have access to personalized discharge care guides, structured pre-discharge education sessions, or scheduled telephonic follow-up support.

The control group caregivers will still undergo a pre-discharge assessment to evaluate their initial knowledge and readiness for discharge. During the follow-up period, their experiences and post-discharge outcomes will be assessed at 7, 15, 30, and 45 days after discharge through surveys and telephone interviews. Data from the control group will serve as a baseline for comparison with the intervention group to determine the effectiveness of the TCG program in improving caregiver preparedness, confidence, and child health outcomes.

The nurse-led TCG program will be developed based on the findings from the qualitative phase and will focus on key areas, such as medication administration, signs of deterioration, feeding techniques, emergency management, and other essential aspects of post-discharge care. The program will also include the provision of a personalized discharge care guide, which will contain instructions on daily care, emergency contact numbers, follow-up schedules, and signs to watch for in the child’s health. Additionally, caregivers in the intervention group will receive telephonic follow-up at 7, 15, 30, and 45 days post-discharge to ensure they have the opportunity to ask questions, reinforce learning, and receive additional support as needed.

The primary outcome measures for this study will include caregiver knowledge of CHD and its management, caregiver readiness for discharge, and caregiver satisfaction with the transition process. Caregiver knowledge will be assessed using a congenital heart disease knowledge scale (CHDKS), which will be developed specifically for this study and validated for reliability and accuracy before data collection. Similarly, caregiver readiness for discharge will be evaluated using a readiness for discharge scale, which will be developed and subjected to rigorous validity and reliability testing. Caregiver satisfaction will be measured through a satisfaction survey assessing their experience with the discharge process, the information provided, and the support received; this tool will also undergo validity and reliability testing before implementation. All research instruments will be carefully developed and tested for content validity, construct validity, and reliability to ensure accurate and meaningful measurement of study outcomes.

Secondary outcomes will include caregiver confidence in managing care tasks, including medication administration and recognizing emergency signs in the child, as well as hospital readmission rates and post-discharge complications within 30 days of discharge. A caregiver confidence assessment tool will be developed specifically for this study and validated for reliability and accuracy before data collection. Hospital readmission rates and post-discharge complications will be tracked using hospital records and follow-up interviews. These outcomes will help assess the broader impact of the nurse-led TCG program on both caregiver preparedness and child health outcomes.

Data collection will be conducted at three time points: baseline (at the time of admission), during discharge, and post-discharge follow-up (starting from seven days after discharge). At baseline (admission), caregiver surveys will be conducted to assess their initial knowledge, readiness, and confidence in managing care. During discharge, post-assessment will be carried out to evaluate changes in caregiver preparedness and understanding after receiving the nurse-led TCG. Post-discharge follow-up, starting from seven days after discharge, will involve caregiver surveys, telephone interviews, and a review of medical records to track hospital readmission rates and post-discharge complications up to 45 days after discharge. The qualitative data will be collected through FGDs, which will be audio-recorded, transcribed, and analyzed using thematic analysis. Quantitative data will be analyzed using statistical methods such as paired t-tests for comparing pre- and post-intervention measures, independent t-tests to compare the intervention and control groups, and multiple regression analysis to identify predictors of caregiver satisfaction and readiness for discharge.

## Results

The primary aim of the study is to assess the effectiveness of the nurse-led TCG program in improving caregiver knowledge, readiness for discharge, and satisfaction with the care transition process. We anticipate that caregivers in the intervention group will show improved knowledge of CHD management, as indicated by higher scores on the CHDKS compared to caregivers in the control group. This improvement is expected to reflect the enhanced education provided through the TCG program, which includes pre-discharge education sessions and a personalized care guide.

Caregiver readiness for discharge is also expected to improve significantly in the intervention group. The readiness for discharge scale will measure how well caregivers feel prepared to manage the child’s care once at home. We hypothesize that caregivers in the intervention group will report higher levels of preparedness due to the additional support provided through the TCG program, which will include structured follow-up and the ability to address any questions or concerns during the post-discharge period.

Additionally, we expect caregiver satisfaction to be significantly higher in the intervention group compared to the control group. The caregiver satisfaction survey will measure satisfaction with the discharge process, the clarity of the information provided, and the level of support received. It is anticipated that caregivers who receive the nurse-led TCG will report greater satisfaction, as the program is designed to address their specific needs and provide continuous support after discharge.

Secondary outcomes will include increased caregiver confidence in managing key aspects of care, such as medication administration and recognizing emergency signs in their child. We expect that caregivers in the intervention group will report greater confidence in these areas, which could translate to a lower likelihood of post-discharge complications and fewer hospital readmissions. Hospital readmission rates and the number of complications within 30 days post-discharge will be tracked as secondary indicators of the effectiveness of the transition care intervention.

Overall, the study is expected to show that the nurse-led TCG program significantly improves caregiver outcomes in terms of knowledge, preparedness, confidence, and satisfaction. Furthermore, it is anticipated that this intervention will result in a reduction in post-discharge complications and hospital readmissions, indicating that better caregiver preparation leads to better child health outcomes during the transition from hospital to home.

## Discussion

This study protocol is designed to evaluate the effectiveness of the nurse-led TCG program in improving post-discharge care for children with CHD and their caregivers. The transition from hospital to home is a critical period for children with CHD, as caregivers often face challenges related to medication adherence, symptom management, emergency recognition, and access to healthcare services. Given the complexity of CHD care, structured interventions that provide comprehensive education, ongoing support, and timely follow-ups are essential in ensuring optimal child health outcomes and caregiver preparedness [8].

Existing literature underscores the significance of nurse-led interventions and structured caregiver education in pediatric populations. Studies have shown that organized discharge planning, follow-up support, and targeted caregiver training can significantly enhance caregiver confidence, reduce anxiety levels, and improve adherence to treatment regimens [9,10]. A systematic review highlighted that nurse-led educational programs focusing on chronic pediatric conditions resulted in better caregiver preparedness, reduced readmission rates, and improved overall child well-being [11]. Furthermore, structured caregiver training models have been linked to enhanced medication adherence and improved management of pediatric health conditions [12].

In addition to in-person training, nurse-led telephone follow-ups and remote support systems have been found to be highly effective in post-discharge care. For instance, a study demonstrated that telephonic follow-ups improved caregiver knowledge, decreased 14-day and 30-day hospital readmissions, and enhanced overall patient satisfaction [13]. Similarly, a randomized controlled trial (RCT) was conducted in Norway, where nurse-led post-discharge telephone support for cardiac surgery patients was associated with better self-management, reduced anxiety, and improved clinical outcomes [14]. These findings support the need for structured follow-up interventions to provide continuous support during the transition period.

Despite the growing evidence on the effectiveness of nurse-led interventions, limited research specifically addresses the unique needs of caregivers managing children with CHD. This study protocol seeks to bridge this gap by designing and testing the nurse-led TCG program, which integrates structured education, post-discharge follow-ups, and tailored support to empower caregivers in managing their child's condition at home [15]. The anticipated findings will contribute to the growing body of evidence on transition care models, reinforcing the need for multifaceted, nurse-led interventions that focus on caregiver empowerment, reduced hospital readmissions, and improved child health outcomes.

By systematically evaluating the impact of this intervention, this study aims to provide a robust evidence base for integrating nurse-led transition care models into routine pediatric CHD management. The findings will inform policymakers, healthcare professionals, and hospital administrators about the importance of structured discharge programs and their role in enhancing long-term pediatric outcomes.

## Conclusions

This study aims to evaluate the effectiveness of a nurse-led transition care guidance (TCG) program for caregivers of children with congenital heart disease (CHD). By utilizing a mixed-methods approach, the protocol focuses on improving caregiver knowledge, confidence, and satisfaction in managing the child's post-discharge care. The expected outcomes include enhanced caregiver preparedness, reduced complications, and improved satisfaction with the transition process. The findings will provide valuable insights into the impact of nurse-led interventions on the care transition for CHD patients, potentially improving both caregiver and patient outcomes.
